# Integrating care for people with mental illness: the Care Programme Approach in England and its implications for long-term conditions management

**DOI:** 10.5334/ijic.516

**Published:** 2010-03-31

**Authors:** Nick Goodwin, Simon Lawton-Smith

**Affiliations:** The King's Fund, 11–13 Cavendish Square, London, W1G 0AN, UK; The Mental Health Foundation, Sea Containers House, 20 Upper Ground, London, SE1 9QB, UK

**Keywords:** care co-ordination, care management, care planning, care programme approach, integrated care, long-term conditions, mental health, personalised care, policy

## Abstract

**Introduction:**

This policy paper considers what the long-term conditions policies in England and other countries could learn from the experience of the Care Programme Approach (CPA). The CPA was introduced in England in April 1991 as the statutory framework for people requiring support in the community for more severe and enduring mental health problems. The CPA approach is an example of a long-standing ‘care co-ordination’ model that seeks to develop individualised care plans and then attempt to integrate care for patients from a range of providers.

**Policy description:**

The CPA experience is highly relevant to both the English and international debates on the future of long-term conditions management where the agenda has focused on developing co-ordinated care planning and delivery between health and social care; to prioritise upstream interventions that promote health and wellbeing; and to provide for a more personalised service.

**Conclusion:**

This review of the CPA experience suggests that there is the potential for better care integration for those patients with multiple or complex needs where a strategy of personalised care planning and pro-active care co-ordination is provided. However, such models will not reach their full potential unless a number of preconditions are met including: clear eligibility criteria; standardised measures of service quality; a mix of governance and incentives to hold providers accountable for such quality; and genuine patient involvement in their own care plans.

**Implications:**

Investment and professional support to the role of the care co-ordinator is particularly crucial. Care co-ordinators require the requisite skills and competencies to act as a care professional to the patient as well as to have the power to exert authority among other care professionals to ensure multidisciplinary care plans are implemented successfully. Attention to inter-professional practice, culture, leadership and organisational development can also help crowd-in behaviours that promote integrated care.

## Introduction

Integrated care is a concept that is becoming widely and more liberally used. It enables an important debate on the future of health and social care provision and stimulates thinking about the range of approaches and models that can be employed to improve care co-ordination across different components of health service delivery. Such debates lie at the heart of current thinking in England about the future management of people with a long-term condition (LTC) where attention has been placed on personalised care planning and active care co-ordination as the key components of integration [1].

This policy paper considers what the long-term conditions policies in England and other countries could learn from the experience of the Care Programme Approach (CPA). The CPA was introduced in England in April 1991 as the statutory framework for people requiring support in the community for more severe and enduring mental health problems [2]. The CPA experience is highly relevant to the current English and international debates on LTC management since it has sought to integrate care and support across primary and secondary health care; across health, social care, welfare, housing and employment support; and across the statutory, independent and voluntary sectors for nearly 20 years.

## Taxonomies of integrated care: where CPA fits

**Figure 1:**
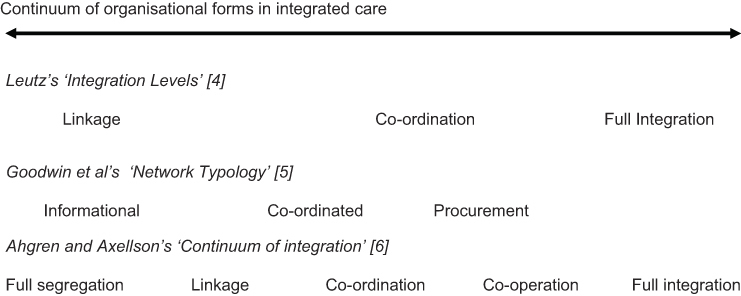
A conceptual continuum of forms of integrated care organisation in health and social care.

There have been several attempts to develop taxonomies of integrated care. Nolte and McKee [3] probably provide the most recent and succinct overview of the most common dimensions of integration that have been put forward, differentiating the concept by type, breadth, degree and process. In addition, several ‘framework’ models have sought to describe the ‘continuum’ of integrated care and how each ‘level’ might better suit a certain care user's need (see Figure 1) [4–6].

Common to each of these ‘framework models’ is the understanding that ‘fully-integrated’ systems—where governance and organisational arrangements are more hierarchical and formal—tend to work best where the needs of the client group are more predictable and well-defined. For example, the relative success of integrated schemes such as PACE (the Programme for All-Inclusive Care of the Elderly) in the USA can be partly attributed to the way elderly clients were pre-selected for their suitability (i.e. they would otherwise be certified to enter a nursing home) and so best benefit from intensive care management by an inter-disciplinary team [7]. For people with less predictable, variable, multiple and/or complex conditions (such as people with long-term mental health problems) the framework models would suggest patients would benefit more from flexible and individually-tailored care based on active care co-ordination across organisational units. A good example is the Canadian PRISMA model that has been a qualified success in co-ordinating care for older and disabled people between organisations through a ‘service continuum’ comprising a single referral entry point, a single assessment and an individual care plan. Service delivery is primarily through contracts with provider agencies since professionals have not generally wished to work directly within the PRISMA system [8, 9].

The essential difference between ‘fully-integrated’ and ‘co-ordinated care’ models is that the former tends to focus on the ‘case management’ of patients with well-defined needs within an institutional setting involving multi-disciplinary teams whilst the latter tends to focus on care management and care brokerage across separate providers of care. Many approaches, of course, are hybrids of these two generalities but CPA in England falls very much into the care co-ordination model—attempting integration for people with severe and long-term mental health problems by knitting together appropriate care from a range of care providers.

## Personalised care planning: CPA in the context of English LTC policy

In England, integrating care for the management of people with a long-term condition (LTC), including the promotion and support of self-care, has become a core Government strategy for its National Health Service (NHS). The detailed business case to redesign provision towards an ‘LTC model’ was first developed in 2004—a vision based primarily on Wagner's Chronic Care Model and the lessons gathered from policy advocate visits to managed care systems in the USA, such as Kaiser Permanente and the Veterans Health Administration [10]. The application of LTC management into practice has since been prioritised by key policies such as the White Paper *Our Health, Our Care, Our Say* [11], the *NHS Next Stage Review* led by Lord Darzi [1] and *World Class Commissioning*—an approach to systematically improving and making more influential the planning, procurement and performance management of the NHS as a key lever in promoting system redesign and improving health and wellbeing [12]. An implicit agenda has been to better co-ordinate care planning and delivery between health and social care as well as to focus on upstream interventions that promote health and wellbeing and minimise illness.

This agenda is more specifically set out in the English Department of Health's mental health strategy for the next ten years: *New Horizons: a shared vision for mental health* [13]. The strategy sets out proposals for improving the mental wellbeing of the population with a specific emphasis on how prevention and early intervention can play a stronger role in mental health care provision where services should ‘work more effectively together’. The strategy recommends the need for higher quality, more personalised mental health services achieved through cross-government and multi-agency commissioning and collaboration.

As a result of these policies a central commitment has been set by the English Government to provide the opportunity for all 15.4 million people with a LTC in England (out of a total population of some 51 million) to have an integrated and personalised care plan by 2010 [1]. Within this lie a number of sub-policies, including the active promotion of self-care strategies to enable people with LTCs to live independently in the home environment (the *Your Health, Your Way* initiative) [14]; the piloting of personal health budgets to enable LTC patients and carers to tailor their care packages [15]; investment in population-oriented health management through the use of predictive-modelling techniques that enable at-risk individuals and populations to be targeted with appropriate interventions [16]; and a movement towards new *Integrated Care Organisations* that potentially provide an in-house set of comprehensive health and social care services to registered patients as well as an advocacy role in brokering the provision of care out with these organisations [17]. The latter has echoes of the medical home models being pioneered in the USA that are the cause of much debate [18].

At present, however, the English Department of Health recognises that the delivery of high quality LTC management is not widespread and requires standardisation across the country. Key issues include: removing barriers to access LTC management; developing the levers and incentives to enable professionals to deliver it; and supporting the workforce to adjust to a new way of working including a focus on governance, professional practice and culture. However, whilst LTC management has become a key priority, relatively little consensus exists on what constitutes best-practice in the management of people with LTCs—specifically on the roles that general practice and other primary, community and social care agencies should play within this. Reviews of the integrated care literature internationally (for example on case management and disease management of people with LTCs) show how our conceptual understanding of the issues varies widely and that understanding what works is a complex and context-specific task [19].

A key underlying philosophy to English health and social care policy is that of ‘personalised care’. Personalised care has been defined in a number of ways, but in essence means a patient-led system that is designed to: promote individual choices in the how, what and where of care; meet an individual's holistic needs through multi-professional assessment and active care co-ordination; and one that promotes patients as equal partners in care, so becoming co-producers of their own health. Personalised care planning appears to be an essential part of the design, yet it is clear that no specific vision yet exists for how this may be delivered. Little guidance, as yet, has been put forward for how a holistic assessment of need should be made; who should take responsibility for the co-ordination of the care package that results; and the skills and leverage that the care co-ordinator needs to be effective. It is likely that much of this guidance may emerge over time as the practical realities of putting policy into practice are appreciated. However, if the roles of different agencies in the process remain ill-defined, there is a danger that LTC management may not become embedded into the way care is provided and/or will not enable a truly holistic or integrated service response.

The development of the CPA model for mental health care, of course, significantly pre-dates this current LTC agenda in England, and the models of care which have inspired it. The lessons from the experience of the CPA model, therefore, are highly relevant if England's LTC policies are to translate effectively into an operational reality. Furthermore, the CPA model may also be a useful template for other countries to consider in designing integrated care services for mental health care and other complex long-term care needs.

## The Care Programme Approach in England

The CPA model in England seeks to integrate care and support services across primary and secondary health care; across health and social care and welfare, housing and employment support; and across the statutory, independent and voluntary sectors. Introduced in 1991, CPA is the statutory framework for people requiring specialist support in the community for more severe and enduring mental health problems [20]. Up to 2008 there were some 485,000 people in England receiving services under CPA (9.5 per 1000 population), although changes to the qualifying criteria in October 2008 mean that now only some 165,000 patients formally receive CPA services (3.2 per 1000 population).

CPA is targeted at adults of working age requiring specialist psychiatric services, such as in-patient care in hospitals, or support from mental health social workers, community psychiatric nurses, counsellors, psychologists and psychiatrists. Such patients will have been diagnosed with a more severe mental disorder such as schizophrenia or bipolar disorder. They may well also have complex needs associated with illicit drug or alcohol misuse, and have experienced multiple admissions to hospital when acutely unwell. Many will be single, unemployed and living alone, and may be reluctant to engage with services.

At its inception, it required Health Authorities, in collaboration with local authority Social Services Departments, to put in place specified arrangements for the care and treatment of mentally ill people in the community. It provided a framework for hospital discharge planning and aftercare, and it was intended that those on CPA should be able to access services 24 hours a day, 365 days a year. Discharge from CPA would happen only when a patient no longer required specialist mental health services or consistently refused services or contact was simply lost.

Specialist community mental health services in England are generally provided by Community Mental Health Teams (CMHTs) serving local areas. These multi-disciplinary teams may consist of community psychiatric nurses, occupational therapists, psychiatrists, psychologists, psychotherapists, social workers, housing workers, welfare rights workers and Support, Time and Recovery (STR) workers who aim to provide companionship and friendship and support with daily living. CMHTs generally support patients with time-limited disorders who are then referred back to their general practitioner (family doctor) when the condition has improved after treatment, though an increasing number provide care for people with chronic and severe mental health problems [21].

Alongside CMHTs are a range of other specialist community teams that have been developed in England over the past ten years. These include Crisis Resolution/Home Treatment Teams, who support people experiencing a crisis at home, to avert a hospital admission; Assertive Outreach Teams, who actively seek to engage with patients living in the community who may be reluctant to use services; and Early Intervention Teams targeting people experiencing a first episode of psychotic illness.

The role of CPA has been to integrate care and support across all these services, and to the wider range of health, social care, welfare, housing and employment services available from both the statutory, voluntary and independent sectors. Hence, CPA is not just about managing a person's specific mental health issue(s) but providing holistic support for their wider needs too.

### The context for the Care Programme Approach

The context for the introduction of CPA in England in 1991 was a mental health system where large asylums either had been, or were in the process of being, closed down and patients transferred either to beds in general hospitals, if it was felt that inpatient care remained necessary, or into the community, generally in residential homes or other types of supported accommodation. From a high of around 150,000 psychiatric beds in the 1950s, by 1990 the number had dropped to some 60,000 (the figure today stands at some 28,000). Under the new arrangements those patients who were moved into the community now required support from a range of organisations across health, social care, welfare benefits, housing, training and employment agencies.

The policy drive towards care in the community was, however, seriously under-funded, with savings from the closure of the asylums often being diverted to other areas of health care. Throughout the 1980s it had become clear that many vulnerable people had been left with little support and were living isolated and sometimes chaotic lives in the community, endangering their own physical and mental health and occasionally that of others.

CPA remained very much at the heart of the New Labour Government's mental health policy when it came to power in 1997:

“A modern mental health service will provide care which is integrated, and which is focused on the individual recognising that different people have different needs and preferences. It will be evidence-based, and outcome driven. Services will be there for people when they need them and where they need them” [22, p. 12].

### Core elements of the Care Programme Approach

At the core of the CPA, and of direct relevance to the English Department of Health's current strategy for LTC management through care planning, are four elements that have stood the test of time for nearly 20 years despite occasional systemic changes:

- a systematic assessment of each patient's health and social care needs;
- the drawing up of a care plan to address those needs;
- the appointment of a ‘key worker’ to oversee the delivery of the care plan; and
- regular review of the patient's needs and care plan.

In 1999, to integrate more effectively health and social care interventions, CPA was amalgamated with local authority Care Management procedures—duties on local authority social services departments to assess needs and purchase services for clients under the NHS and Community Care Act 1990—so that there would be a single process and procedure for people living in the community with enduring mental health needs [22]. This established a system with a single point of referral; a unified health and social care assessment process; co-ordination of the respective roles and responsibilities of each agency in the system; and access, through a single process, to the support and resources of both health and social care.

The main change to the system in the 1999 reform was the introduction of two tiers of CPA. Standard CPA was available for those with lower levels of need and risk. These patients might live independently, have some social support networks of family and friends, be happy to engage with services, and may never have had to be compulsorily detained in hospital. Enhanced CPA would apply to those who had multiple and complex care needs and who were at higher risk, requiring frequent interventions that involved an integrated multi-agency response. Unless there were exceptional circumstances, people detained compulsorily under the Mental Health Act would automatically require Enhanced CPA on discharge from hospital. By 2007/08, there were ∼320,000 people on Standard CPA (6.3 per 1000 population) and 165,000 people on enhanced CPA (3.2 per 1000 population) [23, p. 28].

### The role of the care co-ordinator

The 1999 reform also changed the terminology of ‘key worker’ to ‘care co-ordinator’, although the role remained fundamentally the same. The importance of the role of the care co-ordinator cannot be overstated. It is this person's job to develop the care plan for each individual with their agreement and that of work colleagues; ensure consistency with any specialist service care plans; oversee the delivery of the multidisciplinary care set out in the care plan; measure outcomes; and review plans with patients and colleagues as necessary.

The Care Programme Approach Association (CPAA) issued a Handbook in 2001 [24] offering detailed guidance on the role of the care co-ordinator including the following attributes:

- competence in delivering mental health care (including an understanding of mental illness);
- knowledge of service user/family (including awareness of race, culture and gender issues);
- knowledge of community services and the role of other agencies;
- co-ordination skills; and
- access to resources.

The guidance points out that “The complexity of the care co-ordinator's role in any individual's case will reflect the complexity of that individual's needs. The role is essentially one of co-ordination and communication.” [24, p. 5]. The care co-ordinator will be:

- a qualified professional employed by one of the two statutory services (Health or Social Services) experienced in mental health work;
- generally, the member of the team best placed to fulfil the responsibilities of the role;
- able to co-ordinate assessments that meet the needs of Health and Social Services including risk; and
- able to combine the complimentary roles of care co-ordinator and care manager.

### Care planning

The complexity of the role of the care co-ordinator is reflected in the complexity of care plans. A comprehensive care plan will include both mental health needs, including any clinical care, and physical health needs. It should cover daily living skills, daytime activities including employment, education and training, social and family relationships including the needs of carers and families, finances/welfare benefits, and risk behaviour such as self-neglect or misuse of drugs and alcohol. It should also take into account any needs associated with gender, sexuality, ethnicity and spirituality. Having identified all needs, it then sets out the services that will be provided to meet them, such as a day centre or an employment advice service, and what services they might be able to call on when unwell, such as a Crisis Resolution Team. Although many of these services will be provided by statutory agencies, some may be provided by independent and voluntary sector organisations.

The written care plan, to be co-produced with the patient, should include the name of the care co-ordinator; the support offered; details of where the patient can get help 24 hours a day; what the carer or care co-ordinator should do in the event that a patient's mental health deteriorates rapidly; and arrangements for regular reviews of the care plan with the care co-ordinator.

An example of both the complexity of the CPA arrangements and the intention to provide effective integrated care and support can be found in the CPA Policy issued in March 2007 in North Essex [25]. This document, running to some 65 pages of guidance, practice and protocols, points out that CPA:

“aims to promote effective liaison and communication between agencies, thereby managing risk and meeting the individual needs of those with mental health difficulties so they are better able to function in society… [25, p. 4]. The CPA is an inclusive and dynamic process based on effective communication, appropriate information sharing and negotiation between partners. This negotiation is to draw on available resources to deliver an agreed plan of care, which will provide engagement and involvement from all those involved in the partnership… [25, p. 5]. [The CPA will] ensure service users and carers are involved in the planning to meet their health and social care, leisure, educational and vocational needs…” [25, p. 6].

## Assessing the impact of the Care Programme Approach

It is, of course, one thing to issue detailed guidance on CPA and quite another to ensure that it actually works across a range of front-line services many of which have severe resource constraints that impose huge pressures on staff, and deal with patients who may not only be seriously unwell for long periods but who may not wish to engage with the services offered. One-fifth of service users placed under CPA on discharge from hospital were readmitted within 90 days, suggesting some inability within CPA to keep people safe and supported in the community [26].

CPA has undoubtedly provided many thousands of patients living in the community with an effective framework of care. In a 2006 survey of some 27,000 mental health patients the Healthcare Commission looked at what service users thought of the support they got from the CPA [27]. Overall, over three-quarters (77%) of patients reported their care as good, very good or excellent. This clearly demonstrates that CPA has the potential to provide the holistic integrated care that people need. Yet it has never been consistently or comprehensively implemented, and over the years a number of reports have cited failings in the system. The same survey showed that fewer than half of those on Standard CPA had been given (or offered) a copy of their care plan, and only 70% of those on Enhanced CPA. Of those who had been given or offered a copy of their care plan only 58% said that they definitely understood what was in it. Of those who wanted to be involved in drawing up their plan only 40% said that they definitely had been. Less than half (48%) of those who had wanted information about local support groups had received it. Half (50%) of those who would have liked help in finding work (from mental health services) said they had not received any help, nor had 32% of those who wanted help getting welfare benefits.

The failure to implement the CPA effectively can have tragic consequences, as in one instance cited in a 2008 report by the Mental Health Act Commission (that has a remit to monitor the care given to people subject to the Act):

“A patient discharged into the community without a care plan, support from social services or a place to stay. The patient subsequently left the ward, and was found dead at bottom of a viaduct in a nature reserve” [28, p. 20].

The same Commission report points out that the patchy implementation of the CPA has been a recurring theme of past reports [28, p. 78]. It has also been suggested that some patients with complex needs were nevertheless only being assigned to Standard CPA, effectively meaning CPA was being used as a rationing tool, with some local authorities trying to limit services to both patients and carers to those who are on Enhanced CPA [29]. Effective care co-ordination also requires the development of shared information systems, a long-term NHS and social care priority that has received much investment but has yet to be realised.

Faced with these challenges, the English Department of Health published a consultation paper in 2006, aimed at reforming the CPA process once again in a bid to make it more effective [30]. The paper was honest about the difficulties inherent in the CPA process as it had developed:

“Concern about the loss of the relationship with users of the service was evident throughout. There was disquiet that the CPA has become a managerial tool rather than a system of engaging with people. Also, that the CPA has moved away from the original intention for a system that was mostly designed for people with a serious mental illness that should be used to form a plan of care and treatment and that is a dynamic process that changes through reviews.

It was recognised that there has been inconsistency in implementation and variable standards. Rigidity and inconsistent interpretation were cited as examples of the poor practice. The hypothesis developed that implementation, rather than policy, was at fault with part of the problem being the later changes to the CPA that led to a tick box mentality rather than a proper change process at the beginning with evaluation built in.

Service users expressed concern at the lack of attention to their wider social care needs within their care plan, particularly when the focus has been on problems, risk and subsequent treatment rather than building on their strengths towards recovery. There was equally a concern by service users that not enough attention is paid to contingency or crisis planning. Carers also aired views about their lack of involvement as partners in the care assessment and planning process” [30, p. 2].

## The future of the Care Programme Approach in England

As a result of the consultation, the Department published new guidance on the CPA in March 2008, effective from October 2008 [31]. The guidance abolished the two-tier system of Standard or Enhanced CPA in favour of the original single-tier system. The main aims were to reduce bureaucracy by removing from the formal CPA system those people who have fewer needs that can be met relatively easily (i.e. those on Standard CPA), to refocus CPA on those requiring higher levels of need, especially crisis services, and to establish national competencies for the role of care coordinator role to embed it into the CPA system as a specialist skill.

The guidance highlighted the elements of the whole systems approach that should be followed—an integrated care pathway approach to service delivery; improved information sharing between agencies; protocols and arrangements for working between different assessment and planning systems; improved local shared provider agreements; commissioning for a range of services to meet service users' and carers' needs; and effective multi-agency Local Area Agreements between health and social care commissioners to facilitate planning across agencies. The hope is that a more authoritative and competent cohort of care co-ordinators, working closely and effectively with partners from a range of agencies, will drive through improvements in care for those people who remain under the CPA system.

## Conclusions

### The future for integrated mental health care in England

The number of psychiatric beds in England, currently around 28,000, has been dropping at the rate of around 1500 per year for the past few years. Behind this reduction have been Government policies (such as the creation of Crisis Resolution/Home Treatment Teams, Assertive Outreach Teams and Early Intervention Services) designed to bolster support for people with more severe mental health problems living in the community, thereby reducing hospital admissions. However, with no corresponding reduction in the prevalence rates of mental disorder and current rates expected to remain broadly stable over the next 20 years [32], there is likely to be an increase in the actual numbers of people with a mental disorder in coming years owing to expected rise in the total population of England.

In these circumstances the need for a good system of integrated care for people with chronic mental health needs living in the community is greater than ever, not least because such individuals are also more likely than the general population to have poor physical health as well [33, 34]. The CPA model of integrated care has been remarkably long-lasting. Even with the difficulties it has faced in execution, there has been no serious challenge to its four core elements of a systematic assessment, a care plan, a care co-ordinator and regular review of both needs and the plan.

### Implications for the English LTC programme and other countries

A recent review of integrated care policies in Europe concluded that an active integrated care policy requires that “all actors involved [should] adequately manage dividing lines in the system and the fragmentation of services, such as lack of co-ordination, different professional values and interests” [35]. While the CPA in England may not have been implemented perfectly, the lesson from the CPA experience suggests that there is potential for better care integration to be had in a strategy based on personalised care planning and investment in care co-ordination for people with chronic and sometimes complex needs.

What is clear, however, is that personalised care planning through the CPA or any other model will not reach its full potential unless a number of preconditions are met including: clear eligibility criteria; standardised measures of service quality—based on best practice in patient care; a mix of governance and incentives to hold providers accountable for such quality; and genuine patient involvement in their own care plans.

In terms of the latter point, there remains a political and cultural tension in delivering a traditional government provided mental health service whilst simultaneously championing the role of the individual and their co-ordinator as brokers of how care is delivered and money spent. The English system is in a period of transition towards one where service users are being given the information and leverage to exert personal choices from a competing range of providers (care still delivered free at the point of delivery) *and* in designing their own care package. This is being advanced, for example, through the piloting of personal health budgets and the deployment of direct payments to patients [15].

Of specific concern to the future of LTC management in the English context is that the role of the care co-ordinator appears crucial. Individuals will require such brokers to enable them to make effective decisions about what care they need and are eligible to receive, as well as to champion and co-ordinate what they receive. The CPA experience warns us that care co-ordinators require the skills and competencies to act both as care managers to individual patients (with often very complex and challenging needs) as well as have the power to exert the authority to ensure that care plans are implemented. We know from the evidence, however, that managing across networks of diverse providers to create an integrated care package is problematic because of the lack of power co-ordinators often have to mandate care delivery amongst other agencies [5]. The role requires co-ordinators to be developed as skilled professionals, properly financed and supported, with access to appropriate and timely information. Crucially, the role requires an ability to wield the financial incentives available to gain responsiveness from care providers [5]. Given such lessons, the CPA experience would suggest that the English Department of Health be advised to provide guidance and support to the development of specific care planning and care co-ordination approaches for people with LTCs. At present, this lack of focus on the importance of such a role is a weak link within an overall attractive package of policies.

A final observation from the CPA experience is that integration of care probably needs to concentrate as much on issues of inter-professional practice, culture, leadership, and organisational development as on the systems and the organisational structures that surround it. This finding echoes the findings of work that has examined effective team-working and partnerships where the fostering of a ‘common purpose’ and ‘collective identity’ provide underpinning ideals that facilitate co-operative practice [36].

The English reform process has championed choice, competition and provider diversity and this does not necessarily sit well with the dominant culture that values professional practice and public service. In such an environment of competition, the ability to lever integrated care for people with long-term conditions and long-term mental health problems will rely on mechanisms such as personalised care planning. These need to sit alongside wider commissioning strategies that seek to buy cost-effective and quality-based integrated care services flexible enough to respond to the needs of individual patients with complex needs.

## Reviewers

**Marie Josée Fleury**, PhD, Associate Professor, Department of Psychiatry, McGill University, Adjunct Professor Université de Montréal's Department of Health Administration, Researcher at Douglas Hospital Research Center, Canada

**Susan Gregory**, Dr., Psychiatrist, Hellenic Centre for Mental Health and Research, and Scientific Associate, Department of Health Economics, National School of Public Health, Athens, Greece

**Helen Mildred**, Dr., Senior Clinical Academic, School of Psychology, Deakin University, Burwood, Victoria, Australia
